# Hybrid Tellurium–Lignin Nanoparticles with Enhanced Antibacterial Properties

**DOI:** 10.1021/acsami.0c22301

**Published:** 2021-03-23

**Authors:** A. Gala Morena, Arnau Bassegoda, Javier Hoyo, Tzanko Tzanov

**Affiliations:** Grup de Biotecnologia Molecular i Industrial, Department of Chemical Engineering, Universitat Politècnica de Catalunya, Rambla Sant Nebridi 22, Terrassa 08222, Spain

**Keywords:** lignin, tellurium, hybrid nanoparticles, sonochemistry, antibacterial activity, green synthesis

## Abstract

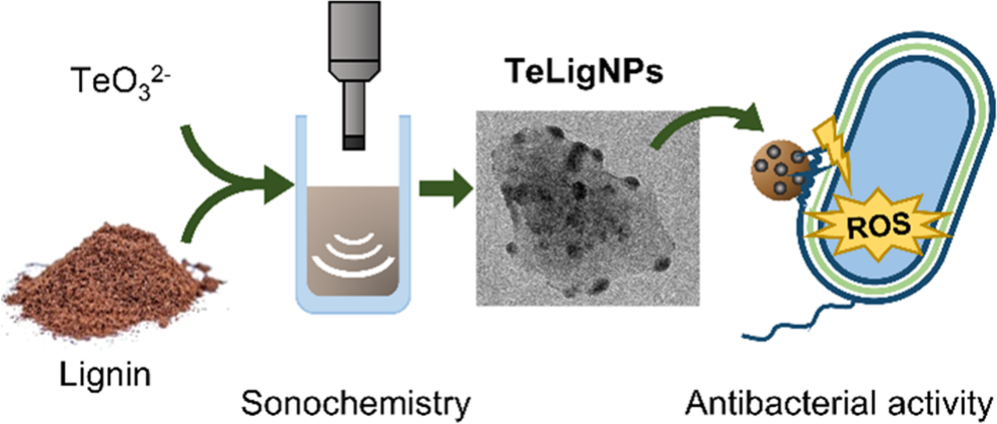

The surge of antibiotic-resistant bacteria is leading to the loss of effectiveness of antibiotic treatment, resulting in prolonged infections and even death. Against this healthcare threat, antimicrobial nanoparticles that hamper the evolution of resistance mechanisms are promising alternatives to antibiotics. Herein, we used Kraft lignin, a poorly valorized polymer derived from plant biomass, to develop novel hybrid tellurium–lignin nanoparticles (TeLigNPs) as alternative antimicrobial agents. The sonochemically synthesized TeLigNPs are comprised of a lignin matrix with embedded Te clusters, revealing the role of lignin as both a reducing agent and a structural component. The hybrid NPs showed strong bactericidal effects against the Gram-negative *Escherichia coli* and *Pseudomonas aeruginosa*, achieving more than 5 log bacteria reduction, while they only slightly inhibited the growth of the Gram-positive *Staphylococcus aureus*. Exposure of TeLigNPs to human cells did not cause morphological changes or reduction in cell viability. Studies on the antimicrobial mechanism of action demonstrated that the novel TeLigNPs were able to disturb bacterial model membranes and generate reactive oxygen species (ROS) in Gram-negative bacteria.

## 1 Introduction

Antimicrobial resistance (AMR) is a naturally occurring phenomenon of bacteria to ensure their survival after exposure to drugs that would normally eliminate them or inhibit their growth. The overuse of antibiotics, however, is exerting selective pressure on bacteria, favoring the surge of drug-resistant strains. As a consequence, the effectiveness of antibiotics for the treatment of bacterial infections has rapidly decreased, resulting in prolonged illness and even death.^1^ The US and Europe have already reached 35 and 25 thousand annual deaths, respectively, related to AMR.^2,3^ If solutions are not found, the global number of deaths per year by 2050 will extend to a dramatic 10 million cases.^4^ Therefore, there is an urgent need to develop alternative and efficient antimicrobials to tackle microbial infections, while preventing the appearance of AMR.

In the search for antibiotic alternatives, metal and metalloid nanoparticles (NPs) are promising antimicrobial agents due to their higher reactivity compared to their bulk counterparts.^5^ Unlike antibiotics, these NPs target multiple bacterial components simultaneously due to their unspecific antimicrobial mode of action including oxidative damage, disruption of the bacterial lipidic membrane, and inhibition of metabolic enzymes, thereby hampering the evolution of resistance mechanisms.^5,6^ Silver is by far the most explored element among the reported antimicrobial metal NPs,^7,8^ while others such as Zn, Cu, and Ti have been less extensively studied.^9,10^ However, findings on the bacterial resistance mechanism against AgNPs^11^ and other metals and metal oxides^12^ define the need to expand the metal NP toolbox against AMR. Despite the known toxicity of tellurite ions (TeO_3_^2–^) against Gram-negative bacteria,^13,14^ the development of Te-based antimicrobial nanomaterials displaying the advantages of metal NPs has only gained interest in recent years.^15−18^ Moreover, toxic reducing agents used in the traditional physicochemical methods for the synthesis of tellurium NPs (TeNPs) are cytotoxic to human cells and environmentally harmful.^19,20^ To overcome the main limitations of traditional synthesis, efforts have been made to develop nanobiotechnological approaches for the synthesis of TeNPs including tellurite-reducing microorganisms^21−23^ and plant-derived reducing agents.^15,16,24^ In these cases, the biomolecules involved in tellurium reduction can act as capping agents, providing enhanced stability of the synthesized TeNPs and increased biocompatibility, thus overcoming the major drawbacks of traditional synthesis methods.^20,25^

In this work, industrial lignin was used for the first time as a reducing agent in a green sonochemical approach to obtain hybrid tellurium–lignin NPs (TeLigNPs). Lignin, the second most abundant biopolymer on Earth, is rarely valorized in its macromolecular form and usually burned for energy uses. Within the context of nanobiotechnology research, lignin is being used for the development of safe-by-design approaches yielding green biocompatible nanomaterials such as porous nanotemplates for infusing silver ions,^26,27^ carriers for drug delivery,^28,29^ and anticancer targeted NPs.^30^

The developed ultrasound-assisted synthesis is a one-pot, fast water-based process, performed under mild conditions and without the need for any chemical modifications of the starting materials. TeLigNPs were extensively characterized in terms of morphology and composition to determine the role of lignin in NP formation. The novel NPs were evaluated as antimicrobial agents by investigating their mode of action and antimicrobial efficacies against Gram-positive *Staphylococcus aureus* and Gram-negative *Pseudomonas aeruginosa* and *Escherichia coli*. Finally, the potential toxicity of the novel TeLigNPs was assessed using human cell models.

## 2 Materials and Methods

### 2.1 Reagents and Cells

Protobind 6000 sulfur-free Kraft lignin powder with an average molecular weight of 1000 g·mol^–1^ was purchased from Green Value (Switzerland). Sodium tellurite, chloroform, phosphate-buffered saline (PBS), Coliform ChromoSelect agar, Cetrimide agar, and Dulbecco’s modified Eagle’s medium (DMEM) were obtained from Sigma-Aldrich (Spain). Nutrient broth (NB) was provided by Sharlab (Spain). AlamarBlue cell viability reagent and molecular probe 2′,7′-dichlorodihydrofluorescein diacetate (H_2_DCFDA) were purchased from Invitrogen, Life Technologies Corporation (Spain). Avanti Polar Lipids provided phosphatidylethanolamine (PE, #840027) and phosphatidylglycerol (PG, #841188) extracted from *E. coli*. Bacterial strains *S. aureus* (ATCC 25923), *E. coli* (ATCC 25922), and *P. aeruginosa* (ATCC 10145), human fibroblast cells (ATCC-CRL-4001, BJ-5ta), and human keratinocyte cells (HaCaT cell line) were obtained from the American Type Culture Collection (ATCC LGC Standards, Spain). The water used in all experiments was purified by the Milli-Q plus system (Millipore) with 18.2 MΩ·cm^–1^ resistivity prior to its use.

### 2.2 Synthesis of TeLigNPs

Protobind 6000 lignin was dispersed in water (1% w/v). To increase lignin solubility, the pH was increased to 9 by addition of NaOH. Thereafter, sodium tellurite powder was diluted in the prepared lignin solution at 100 mM final concentration. The resulting solution was subjected to ultrasound (20 kHz, 50% amplitude, Ti-horn) for 30 min at 60 °C (VCX 750 ultrasonic processor, Sonics). The generated NPs were purified by centrifugation at 25 000*g* for 30 min. The pellet was washed for complete removal of tellurite ions by resuspension in Milli-Q water and were further centrifuged at 25 000*g* for 30 min. Afterward, the washed pellet was resuspended again in Milli-Q water, and low-intensity ultrasound was used to completely disaggregate the NPs in the suspension. Finally, centrifugation at 500*g* for 10 min removed larger particles and the remaining insoluble lignin.

### 2.3 Characterization of TeLigNPs

The morphology and distribution of the TeLigNPs were determined by transmission electron microscopy (TEM) using carbon-coated silicon dioxide grids and a JEOL JEM-2100 LaB6 microscope coupled with energy-dispersive X-ray spectroscopy (EDX) operating at an accelerating voltage of 200 kV. EDX was used for the elemental analysis of the TeLigNPs, and chemical maps were acquired using high-angle annular dark-field (HAADF) scanning TEM (STEM). Nanoparticle size and distribution were obtained using ImageJ software (version 1.52a). The crystalline structures of the TeLigNPs were obtained from high-resolution TEM (HR-TEM) experiments. The ζ-potential of NPs in water was determined using a Zetasizer Nano Z (Malvern Instruments Inc., U.K.).

The content of tellurium in the TeLigNPs was quantified by inductively coupled plasma mass spectrometry (ICP-MS 7800, Agilent Technologies) calibrated by an internal standard with ^45^Rh and a standard curve of ^125^Te. Prior to the analysis, the samples were digested with 20% (v/v) HNO_3_ at 100 °C for 1 h, diluted until a final concentration of 2% HNO_3_, and filtered through a 0.2 μm pore size filter.

### 2.4 Growth Inhibition of *S. aureus*, *E. coli*, and *P. aeruginosa* by TeLigNPs

The antibacterial activity of the TeLigNPs was assessed toward Gram-positive *S. aureus* and Gram-negative *E. coli* and *P. aeruginosa* following the serial dilution method. Overnight bacterial cultures were diluted in NB to an OD_600_ = 0.01 (∼10^5^–10^6^ CFU·mL^–1^). Then, 50 μL of the TeLigNPs at different concentrations (Te content ranging from 0.04 to 2.39 ppm) were mixed with 50 μL of bacterial suspension in 96-well polystyrene plates. The samples were incubated for 24 h at 37 °C with shaking. Bacterial growth in the presence of NPs was assessed by measuring the OD at 600 nm in a microplate reader (Infinite M200, Tecan, Austria). Bacterial inoculum without NPs was used as a growth control (no inhibition). The OD_600_ of the samples at time 0 h was used as a blank. The growth inhibition was calculated as follows
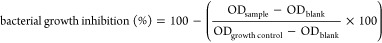
The minimal inhibitory concentration (MIC) was taken as the lowest concentration of TeLigNPs that inhibited the growth of the bacteria after 24 h of incubation at 37 °C. In addition, the number of surviving bacteria during NP treatment was determined after plating 10 μL of the suspensions onto specific agar and further incubation for 24 h at 37 °C. After counting the colonies, the log reduction and the percentage of reduction were calculated.

### 2.5 Bacteria Time-Killing Kinetics

Overnight bacterial cultures were diluted in NB to an OD_600_ = 0.01 (∼10^5^–10^6^ CFU·mL^–1^). For the assay, 300 μL of the bacteria were mixed with 300 μL of TeLigNPs at different concentrations (Te contents 2.39, 1.20, and 0.60 ppm) and incubated at 37 °C with 230 rpm shaking. Samples were taken at different time points, and the surviving bacteria were enumerated using the drop plate method. In total, 10 μL of diluted *E. coli* and *P. aeruginosa* suspensions were plated on Coliform ChromoSelect agar and Cetrimide agar, respectively. After 24 h incubation at 37 °C, the grown colonies were counted. Bacteria in NB were used as a growth control.

### 2.6 Cytotoxicity Test

The cytotoxicity of the TeLigNPs was tested toward human fibroblasts (cell line BJ-5ta) and human keratinocytes (cell line HaCaT). The cells were grown in 100 μL of DMEM in a 96-well plate (60 000 cells per well) at 37 °C in a humidified atmosphere with 5% CO_2_. After 24 h of cell growth, TeLigNPs at different concentrations were incubated with the cells for 24 h. Afterward, the NPs were removed from the wells, and the cells were recovered for 24 h in 100 μL of fresh DMEM. The cell viability assessment was performed using the AlamarBlue assay. AlamarBlue reagent is a redox indicator dye used for the evaluation of the metabolic activity of cells. After removing the culture medium, 100 μL of AlamarBlue reagent diluted in culture medium (10% v/v) was added to each well. After 3 h incubation at 37 °C, the fluorescence was measured (λ_ex_ = 550 nm, λ_em_ = 590 nm). The percentage of cell viability was calculated using the fluorescence values of the wells containing only cells and AlamarBlue reagent as the reference (growth control). Wells containing only AlamarBlue reagent were used as the blank group. The percentage of cell viability was calculated as follows
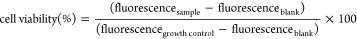
Cell viability was further assessed with fluorescence microscopy using the Live/Dead Viability/Cytotoxicity kit (Thermo Fisher Scientific) that stains the live cells in green and the dead ones in red. After 24 h incubation of the cells with the TeLigNPs, the culture medium was removed and 20 μL of staining solution [0.1% calcein acetoxymethyl (AM) and 0.1% ethidium homodimer-1 in PBS] was added. After 20 min incubation in the dark, the samples were visualized under fluorescence microscopy using a 100× objective lens.

### 2.7 Reactive Oxygen Species (ROS) Generation by TeLigNPs

The reactive oxygen species (ROS) generated by bacterial cultures exposed to the TeLigNPs were evaluated using the oxidation-sensitive probe H_2_DCFDA, which is activated by intracellular oxidants such as hydrogen peroxide and the hydroxyl radical.^13,31^ In the assay, carried out in triplicate, *S. aureus, E. coli*, and *P. aeruginosa* were grown in NB to an OD_600_ of ∼0.8 and were exposed to TeLigNPs (2.39 ppm of Te) for 30 min at 37 °C. The samples were centrifuged at 4000*g* and washed with PBS. The pellet was resuspended in 1 mL of PBS and 1 μL of 20 mM H_2_DCFDA was added. After 30 min incubation in the dark, fluorescence measurements (λ_ex_ = 490 nm, λ_em_ = 520 nm) were performed and normalized to OD_600_. Bacteria incubated without TeLigNPs were used as controls.

ROS generated in human keratinocytes and fibroblasts were assessed after growing the cells in 100 μL of DMEM in a 96-well plate (60 000 cells per well) at 37 °C in a humidified atmosphere with 5% CO_2_. After 24 h, the cells were washed with PBS and exposed to TeLigNPs (2.39 ppm of Te) for 30 min at 37 °C. After removing the particles, 100 μL of 2 mM H_2_DCFDA was added. After 30 min incubation in the dark, fluorescence measurements (λ_ex_ = 490 nm, λ_em_ = 520 nm) were performed. Cells incubated without TeLigNPs were used as controls.

### 2.8 Transmission Electron Microscopy of Bacterial Samples

TeLigNPs were incubated under shaking with *E. coli* and *S. aureus* (OD_600_ = 0.01) for 2 h at 37 °C. The samples were centrifuged at 2000*g* for 5 min at 4 °C and resuspended in a fresh fixative solution containing 2.5% (v/v) glutaraldehyde and 2% (v/v) paraformaldehyde in 0.1 M phosphate buffer, pH 7.4. The samples were incubated with the fixative solution for 1 h at 4 °C, washed three times with 0.1 M phosphate buffer, pH 7.4, and fixed in 1% (w/v) osmium tetraoxide. Afterward, the samples were stained with 2% (w/v) uranyl acetate, dehydrated in ethanol, and embedded in Spurr resin. Ultrathin sections were obtained with an Ultracut E (Reichert-Jung) ultramicrotome and counterstained with lead citrate. Then, the slices were deposited on bare mesh copper grids and the sections were observed using a JEOL 1100 transmission electron microscope at 80 kV.

### 2.9 Gram-Negative Bacteria Model Membranes

A mixture of PE and PG was prepared at an 8:2 (v:v) ratio in CHCl_3_ (0.5 mg·mL^–1^) to mimic a Gram-negative bacterial membrane.^32^ The Langmuir films were formed in a Langmuir trough equipped with two mobile barriers (model KN2002, KSV NIMA, Sweden) mounted on an antivibration table housed in an insulation box at 23 ± 1 °C. Prior to subphase addition (Milli-Q water), the Langmuir trough was cleaned with CHCl_3_ and water, and the surface was further cleaned by suction. Afterward, 40 μL of the lipid mixture solution was added dropwise into the trough filled with the subphase, and after 10 min evaporation of CHCl_3_, the barriers were compressed at 15 cm^2^·min^–1^ until reaching 33 mN·m^–1^, the equivalent of the natural membrane’s lateral surface pressure.^33^ After the stabilization of the membrane for at least 30 min, 500 μL of TeLigNPs in Milli-Q water were inserted beneath the Langmuir film and the changes of the surface pressure derived from their interactions with the already formed bacterial model membrane were recorded. Blank experiments were carried out using the same procedure, but inserting 500 μL of Milli-Q water instead.

## 3 Results and Discussion

### 3.1 TeLigNP Synthesis and Characterization

In the search for antibiotic alternatives against AMR bacteria, antimicrobial metal or metalloid NPs, whose mode of action circumvents the surge of resistance mechanisms, are of high interest for the development of novel antimicrobials. Among them, TeNPs have emerged as promising antimicrobial agents. However, these NPs have been mostly obtained by traditional chemical synthesis yielding TeNPs with low biocompatibility and an associated environmental burden. Previously explored environmentally friendly synthetic approaches mostly rely on the extraction and purification of plant-derived reducing agents and thus, despite being green, are competing with food and healthcare industries.

**Figure 1:**
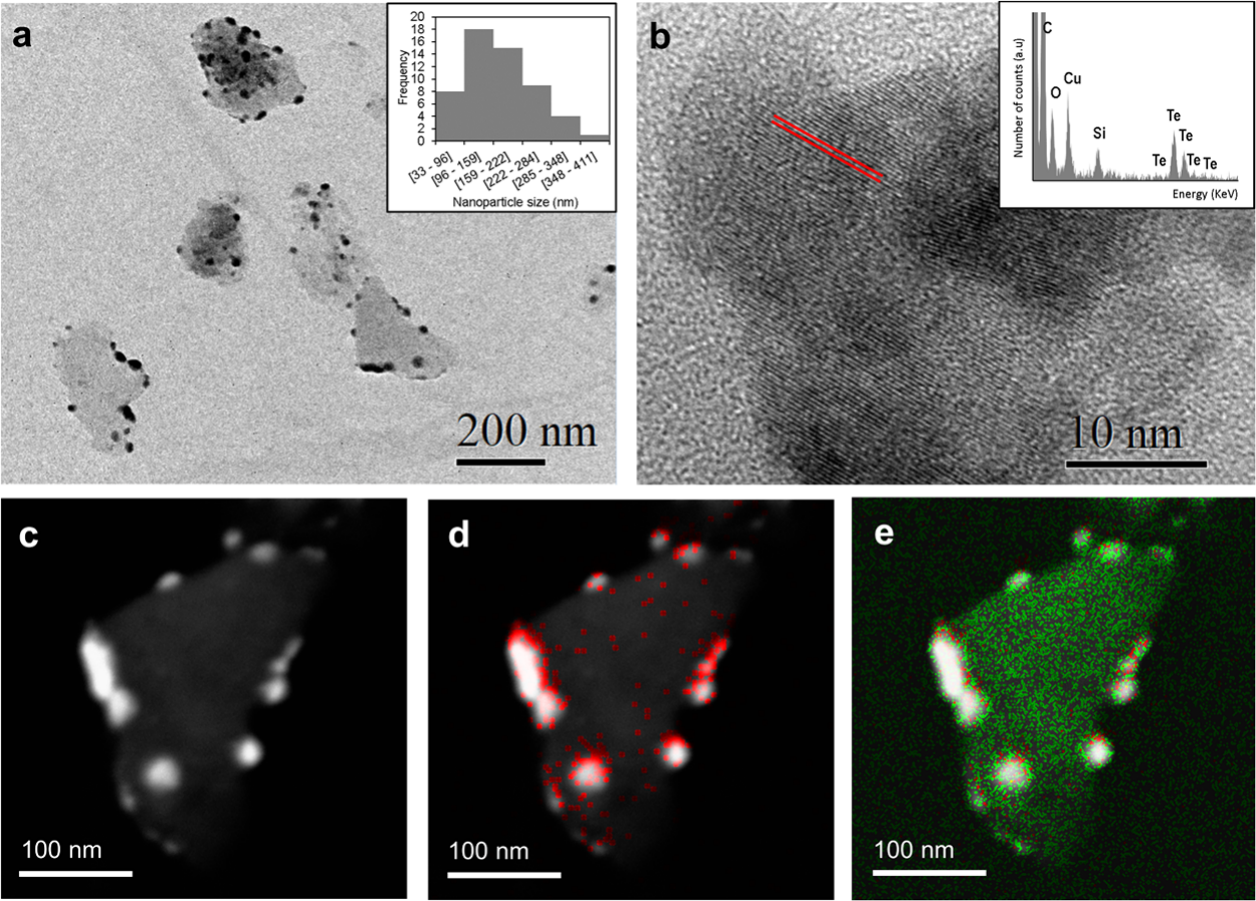
(a) TeLigNP image at 150 000× magnification by TEM and size distribution of TeLigNPs (inset), (b) detailed image of the Te cluster at 500 000× magnification by high-resolution TEM showing the defined crystalline domains (red lines) and EDX spectrum of the clusters (inset), (c) high angle annular dark field scanning transmission electron microscopy (HAADF-STEM) image of TeLigNPs, (d) overlapped HAADF-STEM image with tellurium mapping (red), and (e) overlapped HAADF-STEM image of TeLigNPs with tellurium (red) and oxygen (green) mapping.

Herein, lignin, which is rarely valorized in its macromolecular form, was chosen as a reducing agent for tellurite. This polymer possesses a variety of chemical groups, including phenolic and aliphatic hydroxyl groups,^34^ and it was previously used for reducing silver ions into NPs.^32^ However, initial attempts to reduce tellurite by incubation with a lignin solution did not result in NP formation. To increase the reduction capacity of lignin and increase the NP synthesis yield, a sonochemically assisted approach was adopted. In a high-intensity ultrasound (US) field, reducing species formed from the sonolysis of organic additives and water accelerate the reduction of the metalloid and increase the yield of produced NPs.^35−38^ Moreover, it has been reported that the application of US on lignin causes both side-chain depolymerization and oxidative coupling/polymerization of phenoxy radicals,^39−41^ which in turn may complex with metals.^42^ The darkening of the lignin–tellurite mixture upon application of US suggested the reduction of tellurite to elemental tellurium (Te^0^).^16^ The synthesized NPs were negatively charged, with a ζ-potential of −32.7 mV and an average NP size of 182 ± 85 nm (Figure 1a). The NPs were comprised of an amorphous matrix embedding several electrodense spherical clusters containing defined crystalline domains (red lines in Figure 1b). The elemental map showed that tellurium was mainly located in the electrodense clusters (Figure 1c,d), while the high-intensity oxygen signal observed in the amorphous matrix indicated the presence of lignin containing numerous methoxy and hydroxyl groups (Figure 1e). These images confirmed that in the sonochemical synthesis of TeLigNPs, lignin not only acted as a reducing agent but also as an NP matrix embedding Te clusters.

### 3.2 Antibacterial Activity of TeLigNPs

**Figure 2:**
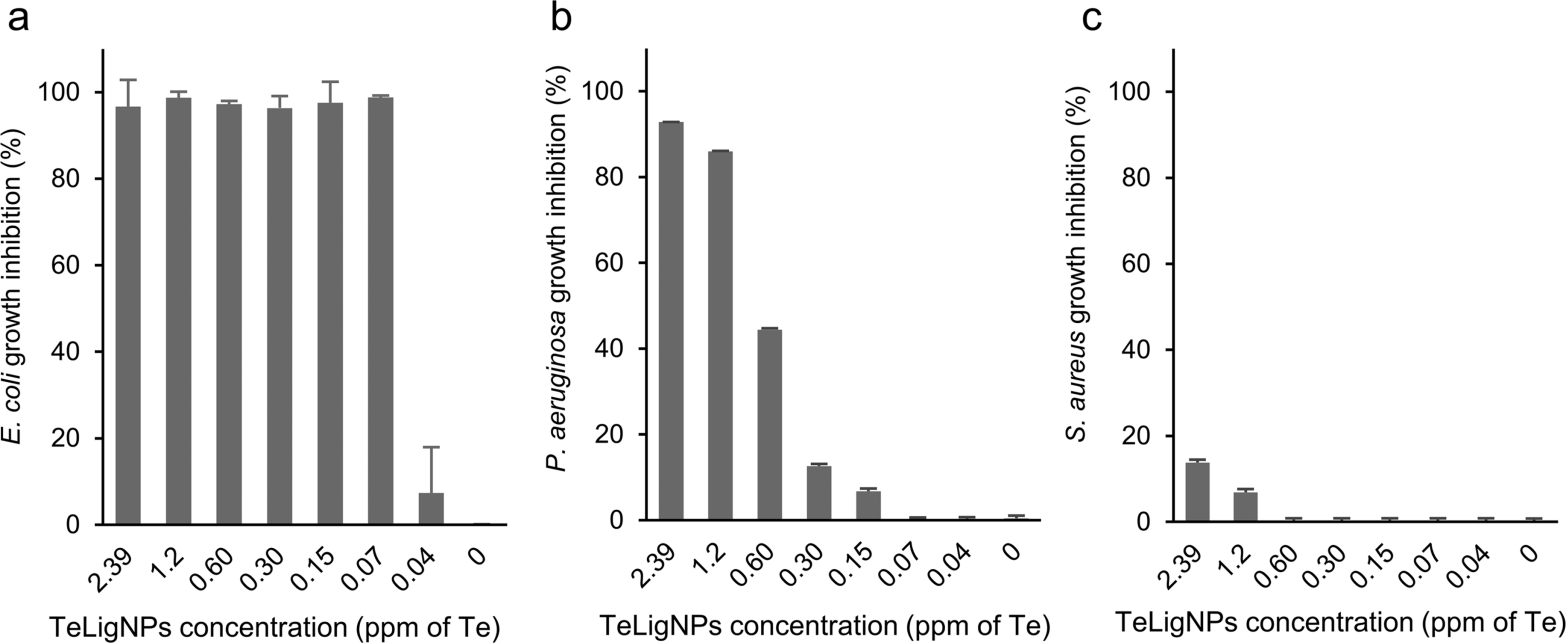
Growth inhibition effect of TeLigNPs against (a) *E. coli*, (b) *P. aeruginosa*, and (c) *S. aureus*. Results are reported as mean values ± SD (*n* = 3).

TeLigNPs at 2.39 ppm of Te were able to completely inhibit the growth of *E. coli* and *P. aeruginosa* (Figure 2a,b), while only 13% growth inhibition could be achieved against the Gram-positive *S. aureus* (Figure 2c). These results are in agreement with previous studies reporting higher antibacterial activity of TeNPs against Gram-negative than Gram-positive bacteria,^16,22,23,43^ a trend also observed for AgNPs.^44^ The evaluation of the MIC of TeLigNPs took into account the concentration of Te, the bactericidal agent in the synthesized hybrid NPs, thus allowing comparison with its bulk counterpart, the tellurite ion (TeO_3_^2–^). While an MIC corresponding to 0.07 and 2.39 ppm of Te was determined for *E. coli* and *P. aeruginosa*, respectively, the same Te concentrations in the form of tellurite did not reduce the growth of these Gram-negative bacteria (Figure S1). Indeed, the MICs of tellurite for *E. coli* and *P. aeruginosa* were 0.31 and 15.60 ppm, respectively (Table S1). These results indicated that the nanoformulation of Te into hybrid TeLigNPs increased the antimicrobial activity of the metalloid.

TeLigNPs showed a strong bactericidal effect against *E. coli* and *P. aeruginosa*, achieving 100 and 99.9995% reduction, respectively, corresponding to more than 5 log reduction (Table 1). To the best of our knowledge, this complete bacterial eradication has not been achieved for previously reported TeNPs, which, in contrast to TeLigNPs, lack a structural polymeric component.^16,22,43^ Thus, these results suggest that lignin not only acts as a green reducing agent but also as a nanoparticle structural component that synergistically enhances the antibacterial activity of the Te clusters.^45^

**Table 1:** Log Reduction and Equivalent Percentage of Reduction (%) of *E. coli* and *P. aeruginosa* Exposed to Different Concentrations of TeLigNPs for 24 h at 37 °C^a^

	*E. coli*		*P. aeruginosa*	
	log reduction	% reduction	log reduction	% reduction
2.39 ppm	6.54 ± 0.00	100.0000	5.42 ± 0.35	99.9995
1.20 ppm	6.05 ± 0.85	100.0000	0.43 ± 0.53	65.2941
0.60 ppm	1.96 ± 1.49	81.7810	0	0
0.30 ppm	2.06 ± 0.14	99.1136	0	0
0.15 ppm	2.26 ± 1.41	97.2455	0	0
0.07 ppm	0.77 ± 0.20	81.8182	0	0

a Results are reported as mean values ± SD (*n* = 3).

**Figure 3:**
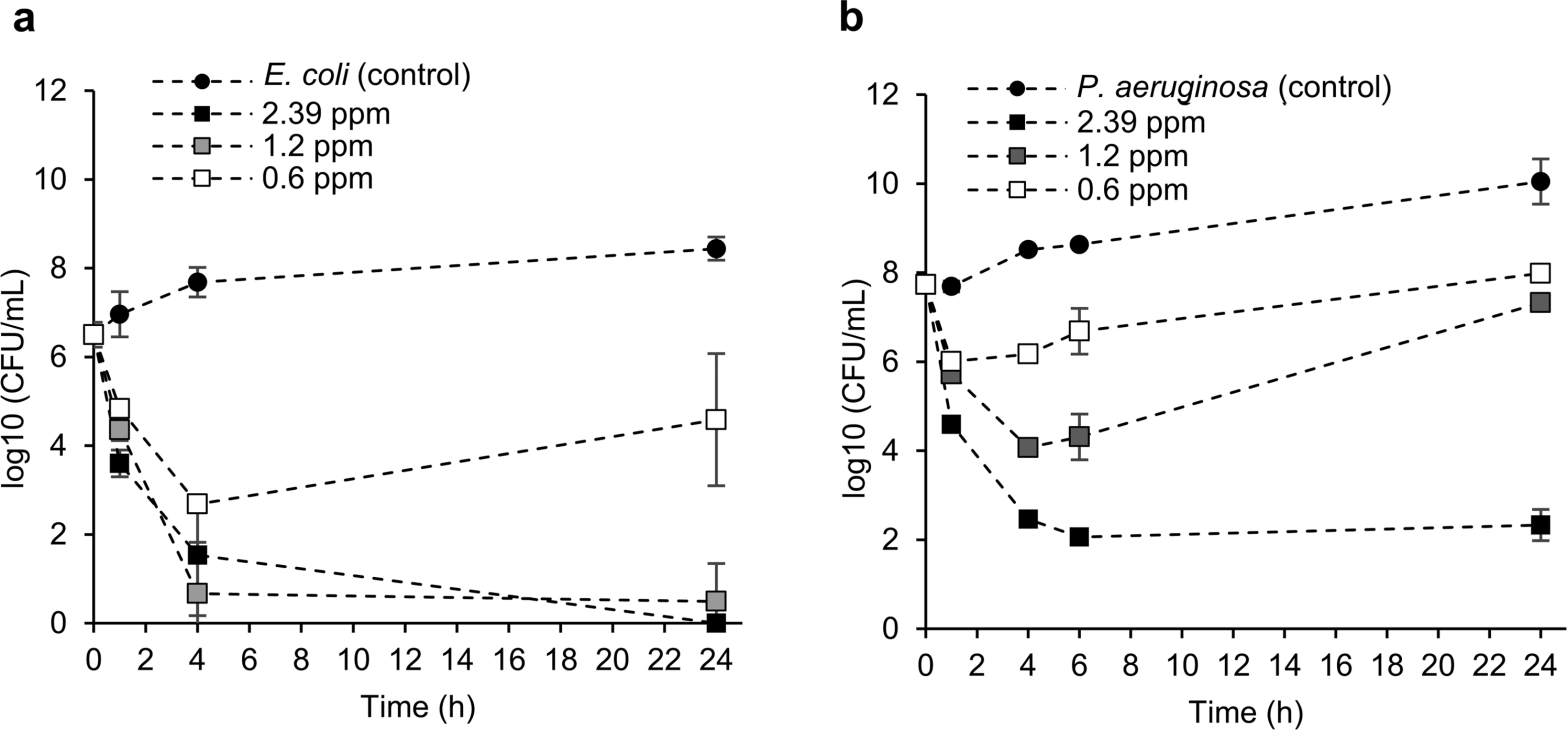
Time-killing curves of TeLigNPs at different concentrations (Te contents 0.6, 1.2, and 2.39 ppm) against (a) *E. coli* and (b) *P. aeruginosa.* Results are reported as mean values ± SD (*n* = 3).

The killing kinetics of the TeLigNPs were assessed to study the bactericidal effect of the hybrid NPs against Gram-negative bacteria as a function of time. The killing curves revealed that TeLigNPs with Te concentrations of 2.39 and 1.2 ppm were bactericidal against *E. coli* within 4 h and achieved complete eradication after 24 h (Figure 3a), in agreement with previous results (Table 1). Regarding *P. aeruginosa*, the bactericidal efficacy of TeLigNPs at a concentration of 2.39 ppm was achieved after 6 h, corresponding to 5.7 log reduction, and was maintained after 24 h (Figure 3b). However, lower concentrations of the NPs exerted only a bacteriostatic effect. Although an initial reduction was observed after 4 h (4 log), *P. aeruginosa* was able to recover and increase up to the initial CFU values.

**Figure 4:**
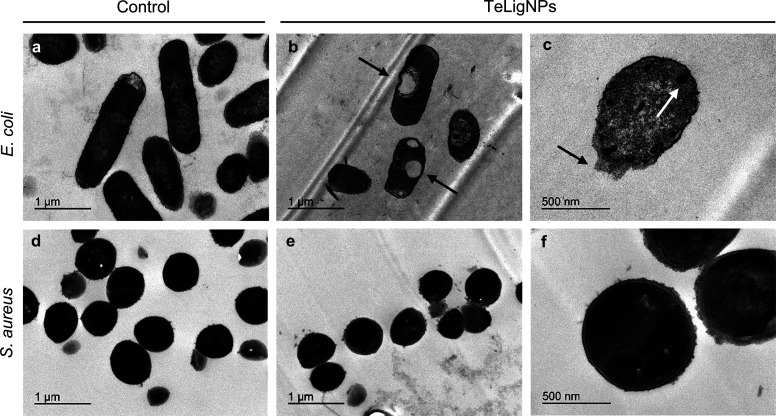
TEM images of *E. coli* (a) before and (b, c) after exposure to TeLigNPs and *S. aureus* (d) before and (e, f) after exposure to TeLigNPs.

An ultrastructural analysis of bacteria was performed to study the morphological changes of the cells exposed to TeLigNPs. A clear difference was observed between control *E. coli* cells and those exposed to the hybrid NPs. Untreated *E. coli* presented a smooth surface and an electrodense cytoplasmic content (Figure 4a), while *E. coli* cells treated with TeLigNPs showed different signs of cellular damage such as loss of cytoplasmic integrity, disrupted cell envelope, and aggregation of intracellular content (arrows in Figure 4b,c). In contrast, *S. aureus* cells exposed to TeLigNPs were undamaged (Figure 4d) and did not present morphological differences compared to the control *S. aureus* cells (Figure 4e,f).

### 3.3 Biocompatibility of TeLigNPs

The biocompatibility of metal- and metalloid-based antibacterial agents is a major issue for biomedical applications in humans. The cytotoxic mechanism of NPs toward eukaryotic cells is associated with oxidative stress, damage to cell membrane and DNA, and consequently apoptosis.^46,47^ These cytotoxic effects are exacerbated when the NPs are chemically synthesized since toxic solvents and reducing agents may remain in the NPs.^20,48^ The TeLigNPs were synthesized following a safe-by-design approach using lignin as a reducing agent. This biopolymer has been innovatively used to reduce silver nitrate for the production of AgNPs,^36,49,50^ to decrease the cytotoxicity of metallic NPs,^27^ and has been formulated in nanocomposites for improving the biocompatibility of biomedical devices.^51^

**Figure 5:**
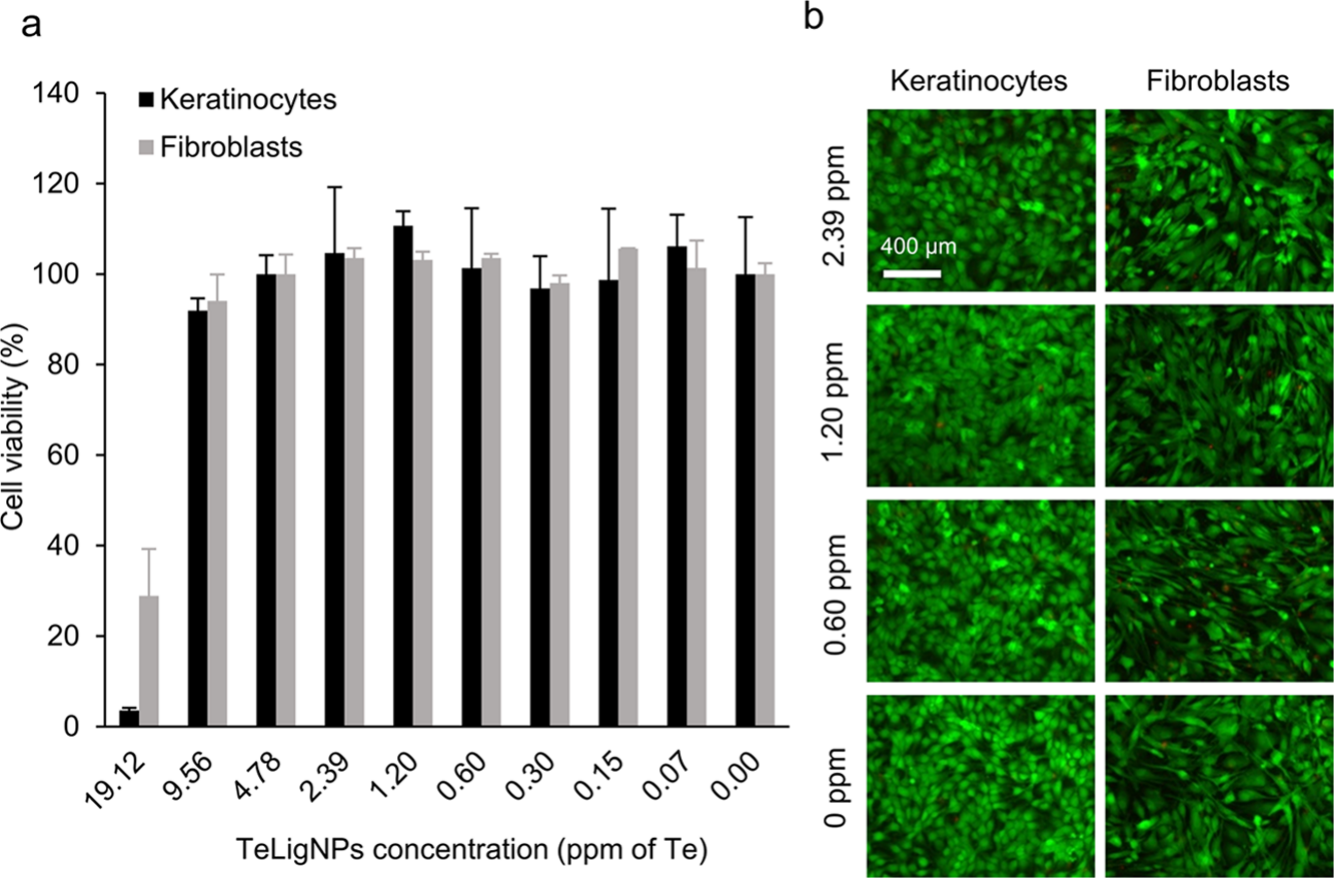
(a) Cell viability (%) of human keratinocytes and fibroblasts treated with different concentrations of TeLigNPs (ppm of Te) for 24 h assessed by the AlamarBlue assay. Results are reported as mean values ± SD (*n* = 3). (b) Live/dead assay of human keratinocytes and fibroblasts treated with different concentrations of TeLigNPs (ppm of Te). The assay stains the live cells in green and the dead ones in red.

The potential toxicity of the novel TeLigNPs was evaluated using human keratinocytes and fibroblasts as cell models. The cell viability of these cell lines upon incubation with tested antibacterial concentrations of TeLigNPs (Te concentrations ranging from 0.07 to 2.39 ppm) did not decrease compared to the untreated cells (Figures 5 and 1a). Remarkably, the cytotoxic effect was only observed on increasing the concentration of TeLigNPs 8-fold (19.12 ppm). Further evaluation by fluorescent microscopy of the cell viability and morphology of human keratinocytes and fibroblasts incubated with TeLigNPs did not reveal cell morphology changes (Figure 5b). The biocompatibility of the TeLigNPs is associated with the presence of a natural, benign organic matrix that embeds the Te clusters.^20^

### 3.4 Mechanism of TeLigNP Antibacterial Action

**Figure 6:**
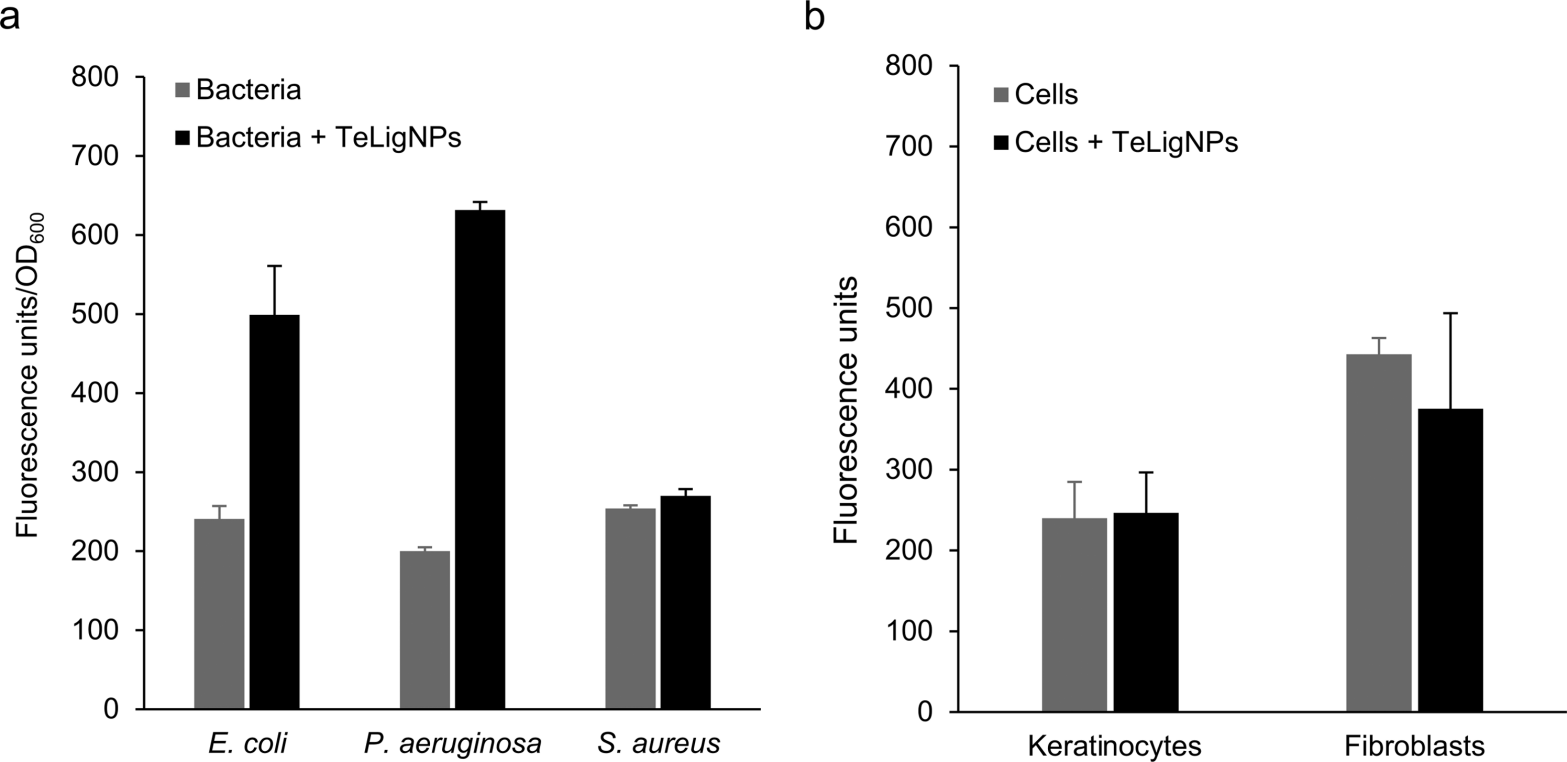
ROS generation assessment using the fluorescent probe H_2_DCFDA after incubation of (a) bacteria (*S. aureus*, *E. coli*, and *P. aeruginosa*) and (b) human keratinocytes and fibroblasts with TeLigNPs. Results are reported as mean values ± SD (*n* = 3).

The bactericidal mechanism of TeNPs has not been completely elucidated, considering the production of ROS as one of the factors involved in their antibacterial capacity.^22^ The toxicity of the tellurium oxyanion in bacteria has been related to superoxide-mediated oxidative stress causing cytoplasmic thiol oxidation, inactivation of iron–sulfur center-containing enzymes, and peroxidation of the membrane lipids, which lead to cell death.^13,52,53^ Therefore, the generation of ROS induced by the TeLigNPs was evaluated to understand the antibacterial mechanism of these hybrid NPs (Figure 6). For both *E. coli* and *P. aeruginosa*, the incubation with TeLigNPs resulted in an increase of the fluorescence emission after the addition of the H_2_DCFDA probe, indicating the presence of ROS induced by the chemical activity of the tellurium oxyanion (Figure 6a). An increase in fluorescence was not detected after incubation of *S. aureus* with TeLigNPs, indicating the absence of cellular damage, in agreement with the previously obtained antimicrobial results (Figure 2). On the other hand, low fluorescence levels detected after incubation of human keratinocytes and fibroblasts with TeLigNPs indicated that these NPs did not induce ROS in the studied cell lines (Figure 6b), in line with the biocompatibility results.

**Figure 7:**
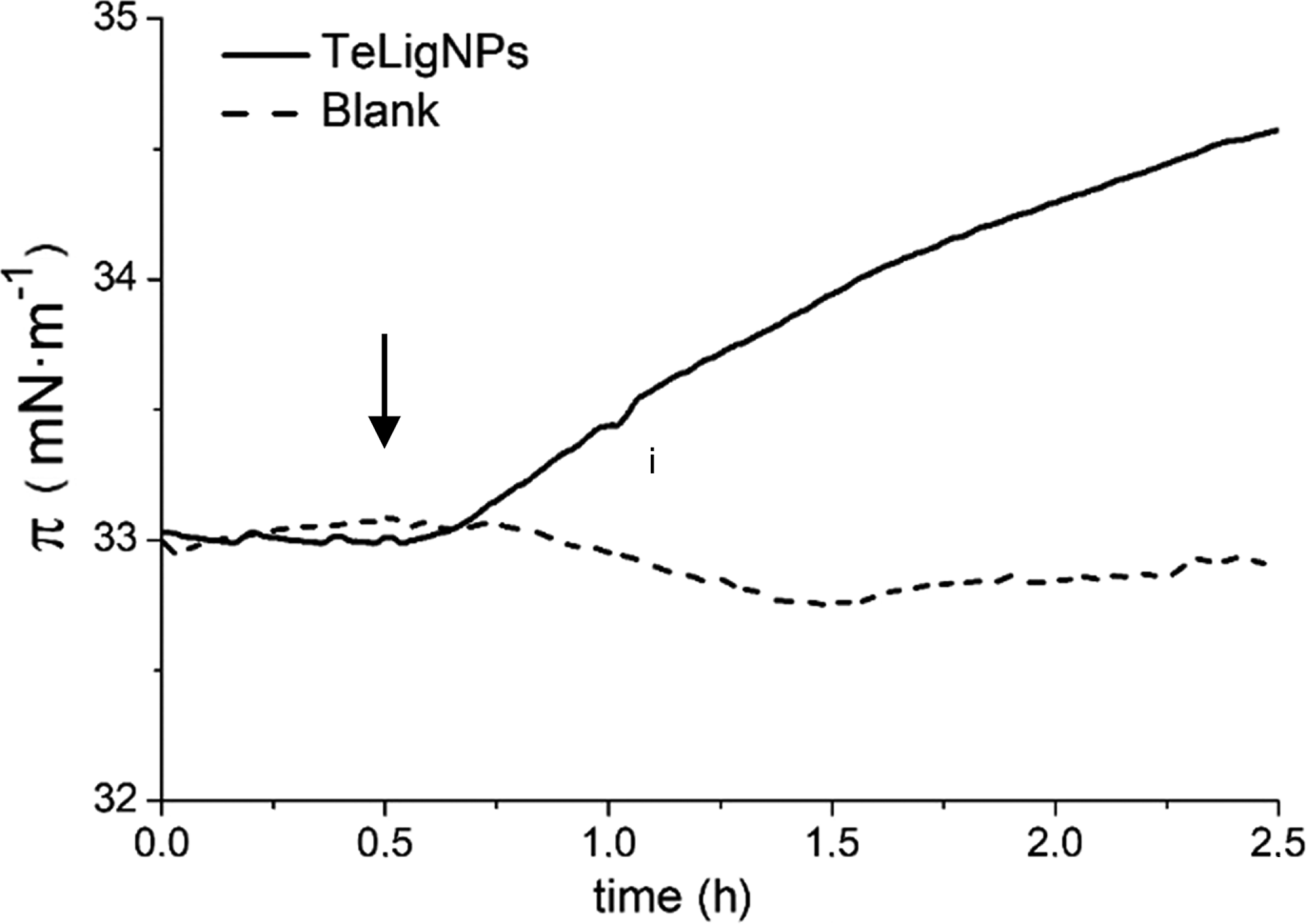
Kinetic adsorption process after the incorporation of the TeLigNPs into the air–water interface of the *E. coli* membrane lipid monolayer at π = 33 mN·m^–1^. The arrow indicates the injection of the TeLigNPs beneath the Langmuir film.

Besides the production of ROS, the ability of TeLigNPs to disturb bacterial membranes was assessed using the Langmuir technique. The injection of the TeLigNPs beneath the prepared Gram-negative model membrane induced an increase of the surface pressure, indicating a membrane-disturbing effect due to the surface activity of the NPs (Figure 7).^54^ These results are supported by the observed irregularities in the cell envelope of the Gram-negative *E. coli* treated with TeLigNPs (Figure 4b,c). Different types of lignin have shown surface activity according to their origin or the processes employed for their purification,^55^ while metallic particles without a stabilizer have shown negligible surface activity. Thus, the observed surface activity was attributed to the lignin component of TeLigNPs, a role also previously described for lignin-capped silver NPs.^56^

Based on the TeLigNP mode of action and the observed different antimicrobial activities against the tested bacteria, we hypothesize that the bactericidal activity against Gram-negative bacteria is due to an insertion of the hybrid NPs into the outer membrane of these bacteria coupled to membrane lipid peroxidation. The surface activity of TeLigNPs would allow binding and insertion into the lipid bilayer of the outer membrane. In this sense, it has been reported that lignin particles adsorb at hydrophilic/hydrophobic interfaces,^57^ and lignin-derived compounds can cross membrane bilayers due to hydroxyl–lipid interactions.^58,59^ Once inserted, ROS production by TeLigNPs generates lipid peroxides that decompose into highly reactive short-chain aldehydes able to diffuse in the cytoplasm and oxidize thiol and amino groups of proteins, thus affecting several cellular processes and leading to death.^60,61^ On the other hand, Gram-positive bacteria are characterized by a thick external peptidoglycan cell wall surrounding the cytoplasmic membrane, which would prevent the access of the TeLigNPs to the cell membrane, thus resulting in the observed low antimicrobial activity against *S. aureus*.

## 4 Conclusions

In this work, we developed novel hybrid antibacterial TeLigNPs through a sonochemical synthetic approach using industrial lignin. This biopolymer was not only used as a tellurite-reducing agent but also became a nanoparticle structural element that embeds reduced Te clusters and confers surface activity to the nanohybrid. The novel NPs completely inhibited the growth of the Gram-negative *E. coli* and *P. aeruginosa* at a lower tellurium concentration than when using the tellurite salt, thus showing the advantages of the applied nanoformulation strategy. Furthermore, the TeLigNPs were found to possess strong bactericidal activity against the aforementioned Gram-negative bacteria, being able to eradicate 99.9995% of these bacteria within 4 h. On the other hand, the hybrid NPs slightly inhibited the growth of Gram-positive *S. aureus*. Investigation of the antimicrobial mode of action revealed the capacity of TeLigNPs to induce a membrane-disturbing effect due to surface activity and the generation of ROS in the presence of Gram-negative bacteria. On this basis, we hypothesize that TeLigNPs enter the lipid bilayer of the outer membrane of Gram-negative bacteria and possibly induce lipid peroxidation. These events cause a disruption of the cell envelope and oxidative damage to several metabolic pathways, eventually leading to cell death. In contrast, the external peptidoglycan cell wall of Gram-positive bacteria prevents the access of the TeLigNPs to the lipid membrane, resulting in the observed low growth inhibition. TeLigNPs did not induce cytotoxic effects or morphological changes to human cell lines, demonstrating that lignin can be used to develop safe-by-design tellurium-based nanomaterials. Altogether, we provided an environmentally friendly approach for preparing biocompatible NPs as a potential antibacterial agent. The capacity of killing bacteria, together with their biocompatibility, makes these NPs promising antimicrobial agents for the treatment of bacterial infections. Further in vivo investigations should be pursued to validate the potential of these NPs for the treatment of infectious diseases.

## Supporting Information Available

Growth inhibition effect of tellurite against *E. coli*, *P. aeruginosa*, and *S. aureus* and MICs of TeLigNPs and tellurite (PDF)

## References

[ref1] WHO. Antimicrobial Resistance: Global Report on Surveillance 2014; World Health Organization, 2014; pp 1–257.

[ref2] CDC. Antibiotic Resistance Threats in the United States; CDC: Atlanta, GA, 2019.

[ref3] ECDC/EMEA Joint Working Group. The Bacterial Challenge: Time to React, EMEA/576176; ECDC, 2009.

[ref4] IACG. No Time to Wait: Securing the Future from Drug-Resistant Infections; IACG, 2019.

[ref5] Slavin Y. N.; Asnis J.; Häfeli U. O.; Bach H. Metal Nanoparticles: Understanding the Mechanisms behind Antibacterial Activity. J. Nanobiotechnol. 2017, 15, 65 10.1186/s12951-017-0308-z. PMC5627441 28974225

[ref6] Gunawan C.; Faiz M. B.; Mann R.; Ting S. R. S.; Sotiriou G. A.; Marquis C. P.; Amal R. Nanosilver Targets the Bacterial Cell Envelope: The Link with Generation of Reactive Oxygen Radicals. ACS Appl. Mater. Interfaces 2020, 12, 5557–5568. 10.1021/acsami.9b20193. 31927911

[ref7] Tang S.; Zheng J. Antibacterial Activity of Silver Nanoparticles: Structural Effects. Adv. Healthcare Mater. 2018, 7, e1701503 10.1002/adhm.201701503. 29808627

[ref8] Zhang X. F.; Liu Z. G.; Shen W.; Gurunathan S. Silver Nanoparticles: Synthesis, Characterization, Properties, Applications, and Therapeutic Approaches. Int. J. Mol. Sci. 2016, 17, 1534 10.3390/ijms17091534. PMC5037809 27649147

[ref9] Azizi-Lalabadi M.; Ehsani A.; Divband B.; Alizadeh-Sani M. Antimicrobial Activity of Titanium Dioxide and Zinc Oxide Nanoparticles Supported in 4A Zeolite and Evaluation the Morphological Characteristic. Sci. Rep. 2019, 9, 17439 10.1038/s41598-019-54025-0. 31767932 PMC6877518

[ref10] Halbus A. F.; Horozov T. S.; Paunov V. N. Strongly Enhanced Antibacterial Action of Copper Oxide Nanoparticles with Boronic Acid Surface Functionality. ACS Appl. Mater. Interfaces 2019, 11, 12232–12243. 10.1021/acsami.8b21862. 30892875

[ref11] Panáček A.; Kvítek L.; Smékalová M.; Večeřová R.; Kolář M.; Röderová M.; Dyčka F.; Šebela M.; Prucek R.; Tomanec O.; Zbořil R. Bacterial Resistance to Silver Nanoparticles and How to Overcome It. Nat. Nanotechnol. 2018, 13, 65–71. 10.1038/s41565-017-0013-y. 29203912

[ref12] Niño-Martínez N.; Salas Orozco M. F.; Martínez-Castañón G. A.; Torres Méndez F.; Ruiz F. Molecular Mechanisms of Bacterial Resistance to Metal and Metal Oxide Nanoparticles. Int. J. Mol. Sci. 2019, 20, 2808 10.3390/ijms20112808. PMC6600416 31181755

[ref13] Pérez J. M.; Calderón I. L.; Arenas F. A.; Fuentes D. E.; Pradenas G. A.; Fuentes E. L.; Sandoval J. M.; Castro M. E.; Elías A. O.; Vásquez C. C. Bacterial Toxicity of Potassium Tellurite: Unveiling an Ancient Enigma. PLoS One 2007, 2, e211 10.1371/journal.pone.0000211. 17299591 PMC1784070

[ref14] Molina-Quiroz R. C.; Muñoz-Villagrán C. M.; de la Torre E.; Tantaleán J. C.; Vásquez C. C.; Pérez-Donoso J. M. Enhancing the Antibiotic Antibacterial Effect by Sub Lethal Tellurite Concentrations: Tellurite and Cefotaxime Act Synergistically in *Escherichia coli*. PLoS One 2012, 7, e35452 10.1371/journal.pone.0035452. 22536386 PMC3334966

[ref15] Gómez-Gómez B.; Arregui L.; Serrano S.; Santos A.; Pérez-Corona T.; Madrid Y. Selenium and Tellurium-Based Nanoparticles as Interfering Factors in Quorum Sensing-Regulated Processes: Violacein Production and Bacterial Biofilm Formation. Metallomics 2019, 11, 1104–1114. 10.1039/c9mt00044e. 31021332

[ref16] Medina Cruz D.; Tien-Street W.; Zhang B.; Huang X.; Vernet Crua A.; Nieto-Argüello A.; Cholula-Díaz J. L.; Martínez L.; Huttel Y.; González M. U.; García-Martín J. M.; Webster T. J. Citric Juice-Mediated Synthesis of Tellurium Nanoparticles with Antimicrobial and Anticancer Properties. Green Chem. 2019, 21, 1982–1998. 10.1039/c9gc00131j. 31156349 PMC6542685

[ref17] Brown C. D.; Cruz D. M.; Roy A. K.; Webster T. J. Synthesis and Characterization of PVP-Coated Tellurium Nanorods and Their Antibacterial and Anticancer Properties. J. Nanopart. Res. 2018, 20, 254 10.1007/s11051-018-4354-8.

[ref18] Lin Y. J.; Khan I.; Saha S.; Wu C. C.; Barman S. R.; Kao F. C.; Lin Z. H. Thermocatalytic Hydrogen Peroxide Generation and Environmental Disinfection by Bi2Te3 Nanoplates. Nat. Commun. 2021, 12, 180 10.1038/s41467-020-20445-0. 33420069 PMC7794375

[ref19] Kus M.; Alic T. Y.; Kirbiyik C.; Baslak C.; Kara K.; Kara D. A. Synthesis of Nanoparticles. In Handbook of Nanomaterials for Industrial Applications; Elsevier, 2018; pp 392–429.

[ref20] Crua A. V.; Medina D.; Zhang B.; González M. U.; Huttel Y.; Miguel García-Martín J.; Cholula-Díaz J. L.; Webster T. J. Comparison of Cytocompatibility and Anticancer Properties of Traditional and Green Chemistry-Synthesized Tellurium Nanowires. Int. J. Nanomed. 2019, 14, 3155–3176. 10.2147/IJN.S175640. PMC6501707 31118629

[ref21] Wang K.; Zhang X.; Kislyakov I. M.; Dong N.; Zhang S.; Wang G.; Fan J.; Zou X.; Du J.; Leng Y.; Zhao Q.; Wu K.; Chen J.; Baesman S. M.; Liao K. S.; Maharjan S.; Zhang H.; Zhang L.; Curran S. A.; Oremland R. S.; Blau W. J.; Wang J. Bacterially Synthesized Tellurium Nanostructures for Broadband Ultrafast Nonlinear Optical Applications. Nat. Commun. 2019, 10, 3985 10.1038/s41467-019-11898-z. 31484932 PMC6726626

[ref22] Zonaro E.; Lampis S.; Turner R. J.; Junaid S.; Vallini G. Biogenic Selenium and Tellurium Nanoparticles Synthesized by Environmental Microbial Isolates Efficaciously Inhibit Bacterial Planktonic Cultures and Biofilms. Front. Microbiol. 2015, 6, 584 10.3389/fmicb.2015.00584. 26136728 PMC4468835

[ref23] Mohanty A.; Kathawala M. H.; Zhang J.; Chen W. N.; Loo J. S. C.; Kjelleberg S.; Yang L.; Cao B. Biogenic Tellurium Nanorods as a Novel Antivirulence Agent Inhibiting Pyoverdine Production in *Pseudomonas aeruginosa*. Biotechnol. Bioeng. 2014, 111, 858–865. 10.1002/bit.25147. 24222554

[ref24] Krug P.; Wiktorska K.; Kaczyńska K.; Ofiara K.; Szterk A.; Kuśmierz B.; Mazur M. Sulforaphane-Assisted Preparation of Tellurium Flower-like Nanoparticles. Nanotechnology 2020, 31, 055603 10.1088/1361-6528/ab4e38. 31618725

[ref25] Singh P.; Pandit S.; Garnæs J.; Tunjic S.; Mokkapati V. R. S. S.; Sultan A.; Thygesen A.; Mackevica A.; Mateiu R. V.; Daugaard A. E.; Baun A.; Mijakovic I. Green Synthesis of Gold and Silver Nanoparticles from Cannabis Sativa (Industrial Hemp) and Their Capacity for Biofilm Inhibition. Int. J. Nanomed. 2018, 13, 3571–3591. 10.2147/IJN.S157958. PMC6016601 29950836

[ref26] Richter A. P.; Brown J. S.; Bharti B.; Wang A.; Gangwal S.; Houck K.; Cohen Hubal E. A.; Paunov V. N.; Stoyanov S. D.; Velev O. D. An Environmentally Benign Antimicrobial Nanoparticle Based on a Silver-Infused Lignin Core. Nat. Nanotechnol. 2015, 10, 817–823. 10.1038/nnano.2015.141. 26167765

[ref27] Nix C. E.; Harper B. J.; Conner C. G.; Richter A. P.; Velev O. D.; Harper S. L. Toxicological Assessment of a Lignin Core Nanoparticle Doped with Silver as an Alternative to Conventional Silver Core Nanoparticles. Antibiotics 2018, 7, 40 10.3390/antibiotics7020040. PMC6023088 29734649

[ref28] Siddiqui L.; Bag J.; Seetha; Mittal D.; Leekha A.; Mishra H.; Mishra M.; Verma A. K.; Mishra P. K.; Ekielski A.; Iqbal Z.; Talegaonkar S. Assessing the Potential of Lignin Nanoparticles as Drug Carrier: Synthesis, Cytotoxicity and Genotoxicity Studies. Int. J. Biol. Macromol. 2020, 152, 786–802. 10.1016/j.ijbiomac.2020.02.311. 32114178

[ref29] Zhou Y.; Han Y.; Li G.; Yang S.; Chu F. Lignin-Based Hollow Nanoparticles for Controlled Drug Delivery: Grafting Preparation Using β-Cyclodextrin/Enzymatic-Hydrolysis Lignin. Nanomaterials 2019, 9, 997 10.3390/nano9070997. PMC6669448 31373282

[ref30] Figueiredo P.; Lepland A.; Scodeller P.; Fontana F.; Torrieri G.; Tiboni M.; Shahbazi M. A.; Casettari L.; Kostiainen M. A.; Hirvonen J.; Hirvonen J.; Teesalu T.; Santos H. A. Peptide-Guided Resiquimod-Loaded Lignin Nanoparticles Convert Tumor-Associated Macrophages from M2 to M1 Phenotype for Enhanced Chemotherapy. Acta Biomater. 2020, S1742–7061, 30561 10.1016/j.actbio.2020.09.038. 33011297

[ref31] Pradenas G. A.; Paillavil B. A.; Reyes-Cerpa S.; Pérez-Donoso J. M.; Vásquez C. C. Reduction of the Monounsaturated Fatty Acid Content of *Escherichia coli* Results in Increased Resistance to Oxidative Damage. Microbiology 2012, 158, 1279–1283. 10.1099/mic.0.056903-0. 22343353

[ref32] Hoyo J.; Torrent-Burgués J.; Tzanov T. Physical States and Thermodynamic Properties of Model Gram-Negative Bacterial Inner Membranes. Chem. Phys. Lipids 2019, 218, 57–64. 10.1016/j.chemphyslip.2018.12.003. 30527783

[ref33] Hoyo J.; Guaus E.; Torrent-Burgués J.; Sanz F. Biomimetic Monolayer Films of Digalactosyldiacylglycerol Incorporating Plastoquinone. Biochim. Biophys. Acta, Biomembr. 2015, 1848, 1341–1351. 10.1016/j.bbamem.2015.03.003. 25771450

[ref34] Lu Y.; Lu Y. C.; Hu H. Q.; Xie F. J.; Wei X. Y.; Fan X. Structural Characterization of Lignin and Its Degradation Products with Spectroscopic Methods. J. Spectrosc. 2017, 2017, 8951658 10.1155/2017/8951658.

[ref35] Pankaj; Ashokkumar M. Theoretical and Experimental Sonochemistry Involving Inorganic Systems; Springer: Netherlands, 2011.

[ref36] Morena A. G.; Stefanov I.; Ivanova K.; Pérez-Rafael S.; Sánchez-Soto M.; Tzanov T. Antibacterial Polyurethane Foams with Incorporated Lignin-Capped Silver Nanoparticles for Chronic Wound Treatment. Ind. Eng. Chem. Res. 2020, 59, 4504–4514. 10.1021/acs.iecr.9b06362.

[ref37] Francesko A.; Cano Fossas M.; Petkova P.; Fernandes M. M.; Mendoza E.; Tzanov T. Sonochemical Synthesis and Stabilization of Concentrated Antimicrobial Silver-Chitosan Nanoparticle Dispersions. J. Appl. Polym. Sci. 2017, 134, 45136 10.1002/app.45136.

[ref38] Okoli C. U.; Kuttiyiel K. A.; Cole J.; McCutchen J.; Tawfik H.; Adzic R. R.; Mahajan D. Solvent Effect in Sonochemical Synthesis of Metal-Alloy Nanoparticles for Use as Electrocatalysts. Ultrason. Sonochem. 2018, 41, 427–434. 10.1016/j.ultsonch.2017.09.049. 29137771

[ref39] Gilca I. A.; Popa V. I.; Crestini C. Obtaining Lignin Nanoparticles by Sonication. Ultrason. Sonochem. 2015, 23, 369–375. 10.1016/j.ultsonch.2014.08.021. 25218770

[ref40] Tortora M.; Cavalieri F.; Mosesso P.; Ciaffardini F.; Melone F.; Crestini C. Ultrasound Driven Assembly of Lignin into Microcapsules for Storage and Delivery of Hydrophobic Molecules. Biomacromolecules 2014, 15, 1634–1643. 10.1021/bm500015j. 24720505

[ref41] Bartzoka E. D.; Lange H.; Thiel K.; Crestini C. Coordination Complexes and One-Step Assembly of Lignin for Versatile Nanocapsule Engineering. ACS Sustainable Chem. Eng. 2016, 4, 5194–5203. 10.1021/acssuschemeng.6b00904.

[ref42] Shimazaki Y. Phenoxyl Radical-Metal Complexes. In PATAI’S Chemistry of Functional Groups; John Wiley & Sons, Ltd: Chichester, U.K., 2012.

[ref43] Pugin B.; Cornejo F. A.; Muñoz-Díaz P.; Muñoz-Villagrán C. M.; Vargas-Pérez J. I.; Arenas F. A.; Vásquez C. C. Glutathione Reductase-Mediated Synthesis of Tellurium-Containing Nanostructures Exhibiting Antibacterial Properties. Appl. Environ. Microbiol. 2014, 80, 7061–7070. 10.1128/AEM.02207-14. 25193000 PMC4249020

[ref44] Xu Z.; He H.; Zhang S.; Wang B.; Jin J.; Li C.; Chen X.; Jiang B.; Liu Y. Mechanistic Studies on the Antibacterial Behavior of Ag Nanoparticles Decorated with Carbon Dots Having Different Oxidation Degrees. Environ. Sci. Nano 2019, 6, 1168–1179. 10.1039/c8en01090k.

[ref45] Espinoza-Acosta J. L.; Torres-Chávez P. I.; Ramírez-Wong B.; López-Saiz C. M.; Montaño-Leyva B. Antioxidant, Antimicrobial, and Antimutagenic Properties of Technical Lignins and Their Applications. BioResources 2016, 11, 5452–5481. 10.15376/biores.11.2.Espinoza_Acosta.

[ref46] Huang Y. W.; Cambre M.; Lee H. J. The Toxicity of Nanoparticles Depends on Multiple Molecular and Physicochemical Mechanisms. Int. J. Mol. Sci. 2017, 18, 2702 10.3390/ijms18122702. PMC5751303 29236059

[ref47] Lewinski N.; Colvin V.; Drezek R. Cytotoxicity of Nanopartides. Small 2008, 4, 26–49. 10.1002/smll.200700595. 18165959

[ref48] Rao C. N. R.; Ramakrishna Matte H. S. S.; Voggu R.; Govindaraj A. Recent Progress in the Synthesis of Inorganic Nanoparticles. Dalton Trans. 2012, 41, 5089–5120. 10.1039/c2dt12266a. 22430878

[ref49] Milczarek G.; Rebis T.; Fabianska J. One-Step Synthesis of Lignosulfonate-Stabilized Silver Nanoparticles. Colloids Surf., B 2013, 105, 335–341. 10.1016/j.colsurfb.2013.01.010. 23399431

[ref50] Hu S.; Hsieh Y. Lo. Silver Nanoparticle Synthesis Using Lignin as Reducing and Capping Agents: A Kinetic and Mechanistic Study. Int. J. Biol. Macromol. 2016, 82, 856–862. 10.1016/j.ijbiomac.2015.09.066.. 26434523

[ref51] Figueiredo P.; Lintinen K.; Hirvonen J. T.; Kostiainen M. A.; Santos H. A. Properties and Chemical Modifications of Lignin: Towards Lignin-Based Nanomaterials for Biomedical Applications. Prog. Mater. Sci. 2018, 233–269. 10.1016/j.pmatsci.2017.12.001.

[ref52] Chasteen T. G.; Fuentes D. E.; Tantaleán J. C.; Vásquez C. C. Tellurite: History, Oxidative Stress, and Molecular Mechanisms of Resistance. FEMS Microbiol. Rev. 2009, 33, 820–832. 10.1111/j.1574-6976.2009.00177.x. 19368559

[ref53] Tremaroli V.; Fedi S.; Zannoni D. Evidence for a Tellurite-Dependent Generation of Reactive Oxygen Species and Absence of a Tellurite-Mediated Adaptive Response to Oxidative Stress in Cells of Pseudomonas Pseudoalcaligenes KF707. Arch. Microbiol. 2007, 187, 127–135. 10.1007/s00203-006-0179-4. 17013634

[ref54] Fernandes M. M.; Ivanova K.; Hoyo J.; Pérez-Rafael S.; Francesko A.; Tzanov T. Nanotransformation of Vancomycin Overcomes the Intrinsic Resistance of Gram-Negative Bacteria. ACS Appl. Mater. Interfaces 2017, 9, 15022–15030. 10.1021/acsami.7b00217. 28393523

[ref55] Barros A. M.; Dhanabalan A.; Constantino C. J. L.; Balogh D. T.; Oliveira O. N. Langmuir Monolayers of Lignins Obtained with Different Isolation Methods. Thin Solid Films 1999, 354, 215–221. 10.1016/S0040-6090(99)00526-X.

[ref56] Hoyo J.; Ivanova K.; Torrent-Burgues J.; Tzanov T. Interaction of Silver-Lignin Nanoparticles With Mammalian Mimetic Membranes. Front. Bioeng. Biotechnol. 2020, 8, 439 10.3389/fbioe.2020.00439. 32457895 PMC7225684

[ref57] Nypelö T. E.; Carrillo C. A.; Rojas O. J. Lignin Supracolloids Synthesized from (W/O) Microemulsions: Use in the Interfacial Stabilization of Pickering Systems and Organic Carriers for Silver Metal. Soft Matter 2015, 11, 2046–2054. 10.1039/c4sm02851a. 25629687

[ref58] Vermaas J. V.; Dixon R. A.; Chen F.; Mansfield S. D.; Boerjan W.; Ralph J.; Crowley M. F.; Beckham G. T. Passive Membrane Transport of Lignin-Related Compounds. Proc. Natl. Acad. Sci. U.S.A. 2019, 116, 23117–23123. 10.1073/pnas.1904643116. 31659054 PMC6859372

[ref59] Boija E.; Johansson G. Interactions between Model Membranes and Lignin-Related Compounds Studied by Immobilized Liposome Chromatography. Biochim. Biophys. Acta, Biomembr. 2006, 1758, 620–626. 10.1016/j.bbamem.2006.04.007. 16733046

[ref60] Pérez J. M.; Arenas F. A.; Pradenas G. A.; Sandoval J. M.; Vásquez C. C. *Escherichia coli* YqhD Exhibits Aldehyde Reductase Activity and Protects from the Harmful Effect of Lipid Peroxidation-Derived Aldehydes. J. Biol. Chem. 2008, 283, 7346–7353. 10.1074/jbc.M708846200. 18211903

[ref61] Wang T. Y.; Libardo M. D. J.; Angeles-Boza A. M.; Pellois J. P. Membrane Oxidation in Cell Delivery and Cell Killing Applications. ACS Chem. Biol. 2017, 1170–1182. 10.1021/acschembio.7b00237. 28355059 PMC5905413

