# Supplementary material for: Hybrid Tellurium–Lignin Nanoparticles with Enhanced Antibacterial Properties
Source: ACS Appl Mater Interfaces. 2021 Mar 23;13(13):14885–93. doi: 10.1021/acsami.0c22301 (PMC8480780; doi:10.1021/acsami.0c22301)
Supplement: Supplementary file 1 — am0c22301_si_001.pdf [file am0c22301_si_001.pdf]

Supporting Information

Hybrid Tellurium-Lignin Nanoparticles with Enhanced Antibacterial Properties

*A. Gala Morena, Arnau Bassegoda, Javier Hoyo, Tzanko Tzanov**

Grup de Biotecnologia Molecular i Industrial, Department of Chemical Engineering, Universitat Politècnica de Catalunya, Rambla Sant Nebridi 22, Terrassa 08222, Spain

Corresponding Author: Tzanko Tzanov (tzanko.tzanov@upc.edu)

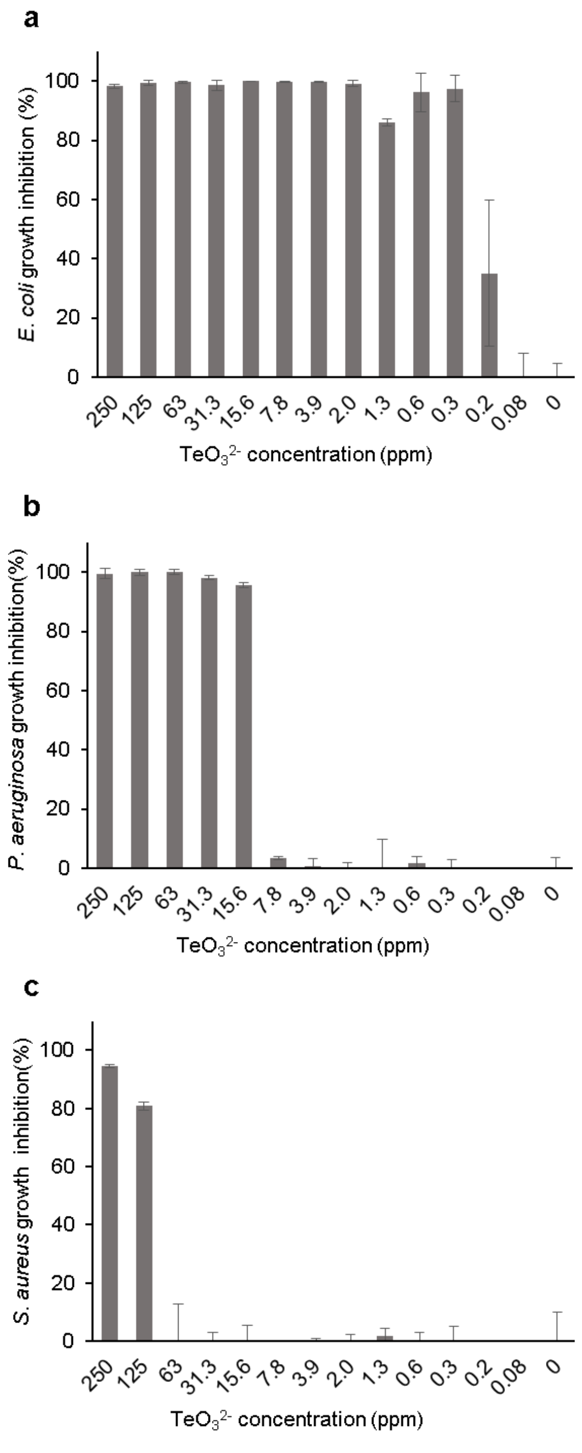

Figure S1. Growth inhibition effect of tellurite (TeO_3^{2-}) against (a) *E. coli*, (b) *P. aeruginosa* and (c) *S. aureus*. Results are reported as mean values ± SD (n = 3).

Table S1. Minimum inhibitory concentration (MIC) values of TeLigNPs and tellurite ion (TeO_3^{2-}) (ppm) assessed toward *E. coli, P. aeruginosa* and *S. aureus*.

	TeLigNPs	TeO_3^{2-}
E. coli	0.07	0.31
P. aeruginosa	2.39	15.60
S. aureus	> 2.39	250.00
